# Understanding rice adaptation to varying agro-ecosystems: trait interactions and quantitative trait loci

**DOI:** 10.1186/s12863-015-0249-1

**Published:** 2015-08-05

**Authors:** Shalabh Dixit, Alexandre Grondin, Cheng-Ruei Lee, Amelia Henry, Thomas-Mitchell Olds, Arvind Kumar

**Affiliations:** International Rice Research Institute, DAPO Box 7777, Metro Manila, Philippines; Department of Biology, Duke University, Durham, NC 27708 USA; Present address: Department of Agronomy and Horticulture, University of Nebraska-Lincoln, Lincoln, NE 68583 USA; Present address: Gregor Mendel Institute of Molecular Plant Biology, Vienna, Austria

**Keywords:** Rice, Drought, Yield, Lodging, Direct seeding, QTL

## Abstract

**Background:**

Interaction and genetic control for traits influencing the adaptation of the rice crop to varying environments was studied in a mapping population derived from parents (Moroberekan and Swarna) contrasting for drought tolerance, yield potential, lodging resistance, and adaptation to dry direct seeding. A BC_2_F_3_-derived mapping population for traits related to these four trait groups was phenotyped to understand the interactions among traits and to map and align QTLs using composite interval mapping (CIM). The study also aimed to identify QTLs for the four trait groups as composite traits using multivariate least square interval mapping (MLSIM) to further understand the genetic control of these traits.

**Results:**

Significant correlations between drought- and yield-related traits at seedling and reproductive stages respectively with traits for adaptation to dry direct-seeded conditions were observed. CIM and MLSIM methods were applied to identify QTLs for univariate and composite traits. QTL clusters showing alignment of QTLs for several traits within and across trait groups were detected at chromosomes 3, 4, and 7 through CIM. The largest number of QTLs related to traits belonging to all four trait groups were identified on chromosome 3 close to the *qDTY*_*3.2*_ locus. These included QTLs for traits such as bleeding rate, shoot biomass, stem strength, and spikelet fertility. Multivariate QTLs were identified at loci supported by univariate QTLs such as on chromosomes 3 and 4 as well as at distinctly different loci on chromosome 8 which were undetected through CIM.

**Conclusion:**

Rice requires better adaptation across a wide range of environments and cultivation practices to adjust to climate change. Understanding the genetics and trade-offs related to each of these environments and cultivation practices thus becomes highly important to develop varieties with stability of yield across them. This study provides a wider picture of the genetics and physiology of adaptation of rice to wide range of environments. With a complete understanding of the processes and relationships between traits and trait groups, marker-assisted breeding can be used more efficiently to develop plant types that can combine all or most of the beneficial traits and show high stability across environments, ecosystems, and cultivation practices.

**Electronic supplementary material:**

The online version of this article (doi:10.1186/s12863-015-0249-1) contains supplementary material, which is available to authorized users.

## Background

Rice growing environments are highly diverse and are affected by fluctuations in environmental conditions during crop growth. Water shortages due to climate change, increased competition for fresh water from industries and domestic usage, and increasing labor and fertilizer costs threaten the sustainability of the transplanted system of rice cultivation [1–3]. With reduced water availability, rice cannot be kept flooded for its entire duration and field conditions vary frequently from anaerobic to aerobic across the season. Such situations demand a better adaptation of rice to variable conditions. In a slow but steady manner, rice is moving towards adaptation to new cultivation practices such as direct seeding (in non-puddled and puddled soil), alternate wetting and drying, and non-puddled transplanted rice cultivation systems which are likely to be predominant in the future. While such water-saving technologies have their benefits, there are several associated risks. For example, shifting rice cultivation from continuous flooding to variable anaerobic to aerobic cycles affects yield. This is primarily due to the exposure of the crop to mild water stress [4] and due to the reduced nutrient uptake under non-flooded aerobic conditions [2]. Rice varieties specifically developed for flooded transplanted conditions show variable degrees of yield decline, depending upon the period during which they are exposed to non-flooded aerobic conditions. Apart from this, aerobic conditions lead to other problems such as non-uniform establishment and increased weed pressure. Irregular shifts from anaerobic to aerobic conditions require rice roots to adapt quickly to maintain water and nutrient uptake and utilization. Under such frequently changing conditions, traits related to early and uniform establishment, maintenance of growth rate at vegetative stage, and efficiency to successfully complete the reproductive and grain-filling phase determine the yield stability. Along with this, traits such as yield potential and resistance to lodging also play a role in determining yield stability by ensuring higher yield and minimum yield loss.

To adapt to such variable conditions, ideally the plant should possess a combination of morpho-physiological traits such as high yield potential, early and uniform emergence, better weed competitiveness, and lodging resistance [2]. Apart from this, root traits leading to better water and nutrient uptake, tolerance to mild to moderate drought, and resistance/tolerance to prevalent biotic stresses also play a crucial role. Recent studies have examined the adaptation of rice to aerobic conditions [5–9], yield and yield-related traits [10–13], and lodging resistance [14, 15]. These studies provide detailed accounts of targeted traits, and the majority of them target the trait groups separately. However, understanding the genetic control of adaptability and productivity of rice across variable environments and cultivation practices demands a more elaborate approach. Morphological characteristics that may appear unrelated at the phenotypic level may be affected by the same or related physiological response or may have related genetic control. Studying a wide range of factors affecting different morphological and adaptive characteristics can provide better insight into these interactions. Genomic regions, particularly those that affect a wide range of traits, also need to be identified for use in marker-assisted breeding. To address these aspects collectively, this study was conducted to describe the relation and the genetic basis of four diverse trait groups: drought tolerance, yield potential, lodging resistance, and adaptation to direct seeding, their interactions and the genetics behind them on a mapping population derived from two parents that are contrasting in all four trait groups. Component traits were studied individually and as composite traits to provide a clearer understanding of the genetic control of rice adaptation to varying environments. The study identified major QTLs and QTL clusters related to these traits using composite interval mapping (CIM) and multivariate least square interval mapping (MLSIM) to identify loci that affect the four trait groups as composite traits and determine the proportion of effect of each component trait to the multivariate QTLs.

## Results

### Parental diversity

**Fig. 1:**
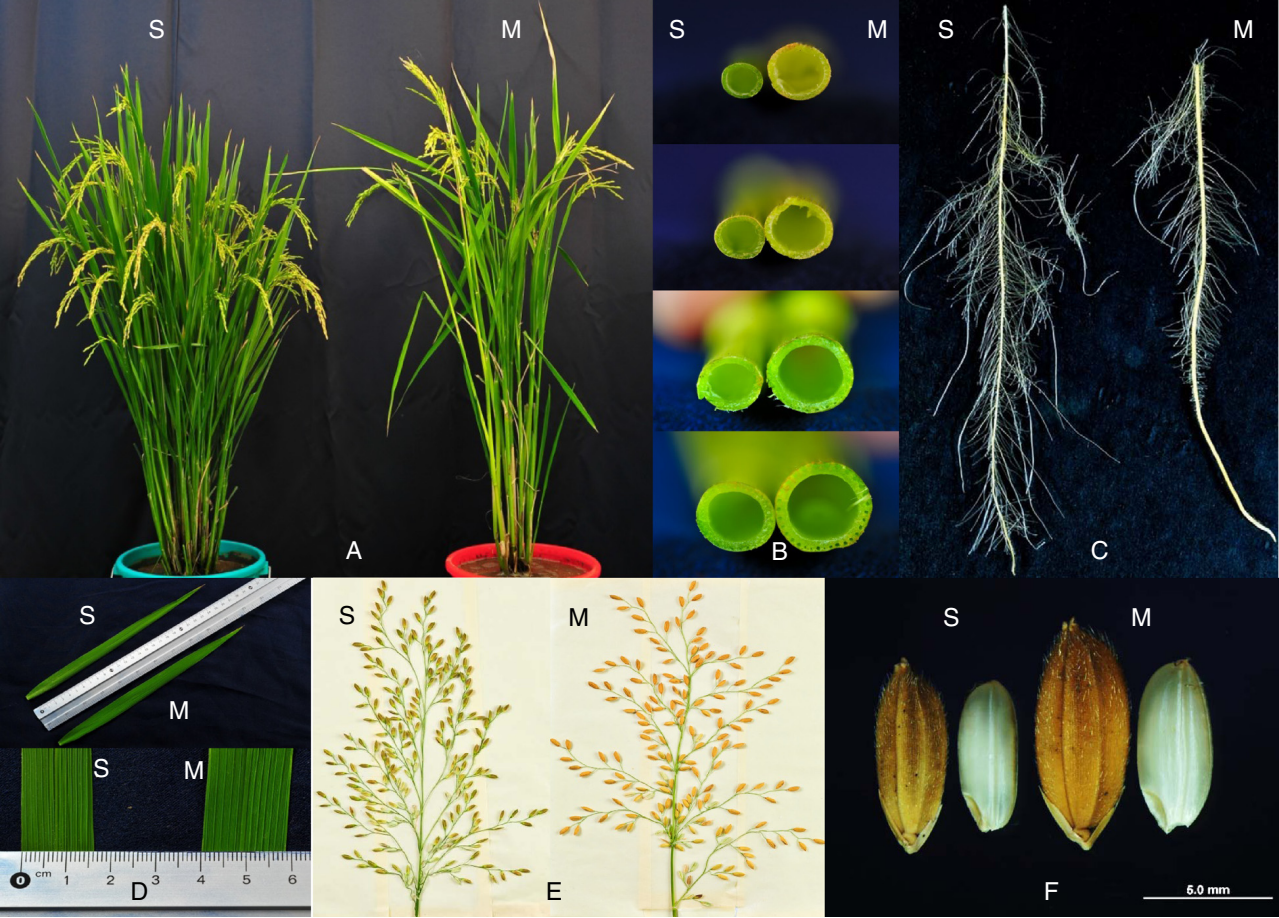
Morphological differences between rice cultivars Swarna (S) and Moroberekan (M). **a** Plant type, tiller and panicle number; **b** Stem diameter (first to fourth internode from the bottom); **c** Root architecture at seedling stage; **d** Flag leaf length and width. **e** Panicle architecture and grains per panicle; **f** Grain type and size

The cultivars Moroberekan and Swarna showed high contrast for plant type and other key traits that determine performance in terms of the four trait groups considered in this study (Fig. 1). Additional file 1 presents the differences between Moroberekan and Swarna for some of the key traits that determine the morphology and yield of rice under varying environmental conditions. Swarna, being the high-yielding parent, showed higher values for yield and for traits related to yield potential such as tiller and panicle number in all three environmental conditions (well-watered, drought, and direct-seeded). However, the higher tolerance of Moroberekan to drought allowed it to maintain higher spikelet fertility compared to Swarna under severe drought stress. The yield decline under drought stress (compared to the non-stress treatment) in Swarna was much higher as compared to Moroberekan, showing the higher susceptibility of Swarna to drought. Swarna showed quicker emergence and a higher number of nodal roots while Moroberekan showed a higher percentage of deep roots under lowland drought at maturity. However, Swarna showed higher root mass density at shallow depths and Moroberekan showed higher stem diameter and sturdiness.

### Phenotypic variation within the progeny

**Fig. 2:**
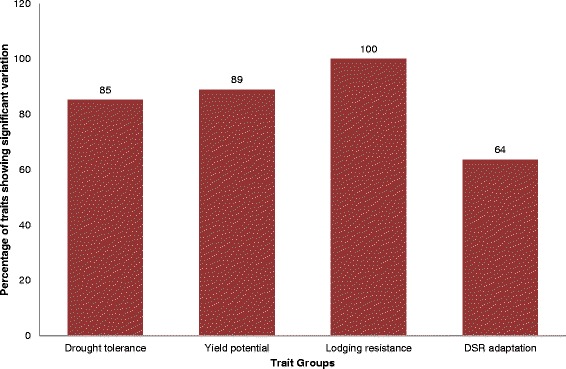
Percentage of traits showing significant variation in ANOVA across the four trait groups

The phenotypic variation among the parents was transferred to the progeny and significant genetic variation for a large proportion of traits studied under each trait group was observed. Significant differences among the progeny for 64–100 % of the traits were observed for the different trait groups (Fig. 2). Additional files 2, 3, 4 and 5 present the results of the analysis of variance (ANOVA) conducted for all experiments. In the three experiments conducted under lowland drought, a higher yield decline for parents and progenies was observed in Experiment 1A as compared to 1B and 1C. The progenies showed significant variation for all traits except for Normalized Difference Vegetation Index (NDVI), reduction of NDVI, root mass density, and percentage deep roots for which consistency in significance was not observed across the three maturity groups (Additional file 2). The progeny means ranged between the parent means or were equal to one of the parents for most traits except for some traits such as bleeding rate (in Experiment 1A and 1B), root mass density at 15–30 cm (in Experiment 1A), and days to flowering (in the three experiments). Under well-watered lowland conditions (Experiment 2), significant variation for all traits except spikelet fertility was observed (Additional file 3). The specificity of significance of variation for spikelet fertility under drought stress showed the higher level of tolerance to drought of Moroberekan over Swarna. Similar to the stress conditions, the progeny mean of the majority of the traits ranged between the two parents or were equal to one of the parents with the exception of days to flowering which stayed lower than both parents (Additional file 3). For lodging-related traits, significant differences were observed for all traits under both lowland and upland well-watered conditions (Additional files 3 and 4). The progeny means were intermediate for traits such as plant height, stem diameter, and stem strength with Moroberekan on the higher and Swarna on the lower side. These three traits played a crucial role in determining the resistance to lodging of the progeny with dwarf plant stature, larger stem diameter, and higher stem strength leading to higher resistance to lodging. The population was also screened under upland dry direct-seeded conditions to determine their adaptation to direct seeding (Experiments 3 and 4). Under well-watered upland conditions, significant variations for all traits except spikelet fertility were observed (Additional file 4). However, under seedling stage drought conditions, significant differences for early and uniform establishment were not observed (Additional file 5).

### Trait correlations and interaction between trait groups

**Fig. 3:**
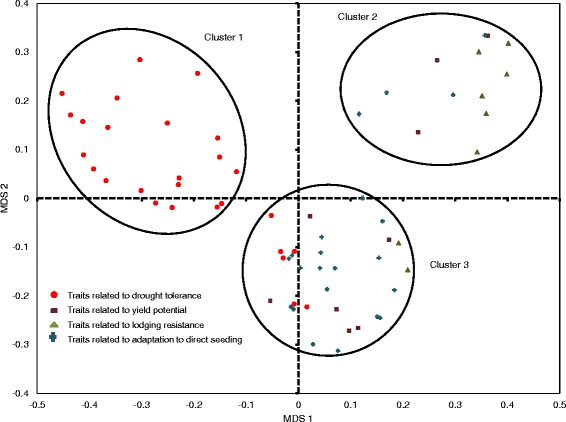
Multi-dimensional scaling (MDS) analysis conducted using the correlation matrix of 66 traits belonging to the four different trait groups

**Fig. 4:**
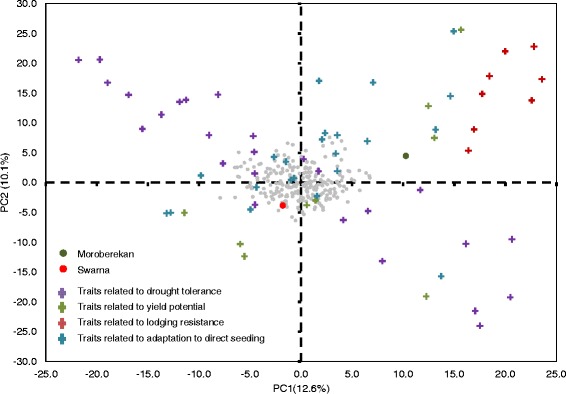
PCA on trait correlations in parents and progeny for the four trait groups. Gray dots represent the genetic means of each progeny; Red and green circles represent means for Swarna and Moroberekan, respectively. Crosses (color coded as presented in the legend) indicate the loadings for each trait along the first two components, which comprise 22.7 % of the total genetic variation for all traits

Correlations among the traits belonging to the four different trait groups are presented in Additional file 6. The analysis showed higher levels of correlations within trait groups as compared to those across trait groups. In general, higher levels of correlation were observed between traits related to yield potential, lodging resistance, and adaptation to direct seeding while drought tolerance-related traits showed lower correlation with the other three trait groups. The multidimensional scaling (MDS) analysis divided the traits into three distinct clusters based on the correlations between them (Fig. 3). Cluster 1 specifically constituted of drought-related traits, cluster 2 contained most of the lodging-related traits and some traits related to yield potential and adaptation to direct seeding, and cluster 3 contained correlated traits across all four trait groups. Most of the traits related to adaptation to direct seeding belonged to this cluster. Interestingly, some of the root-related traits measured under drought stress grouped with cluster 3, showing the importance of these traits under direct-seeded conditions. Principal component analysis (PCA) was conducted to further examine the relationships among traits. The first two components together explained 22.7 % of the genetic trait variation, showing a mild level of genetic correlation among the traits (Fig. 4). Components 1–8 together explained 50.1 % of the variation while components 1–20 explained 75.5 % of the variation (Additional file 7). This can be attributed to the large number and diversity of traits. The PCA may explain higher percentage variations if traits belonging to each trait group are analyzed separately. However, analyzing them together allowed us to view the pattern of arrangement for all four trait groups simultaneously on PC1 and PC2. The PCA further resolved the trait groups along the two axes, and a clearer grouping of traits within each trait group was observed. The progenies were distributed almost evenly across the four quadrants; however, a large difference in the positioning of parents Moroberekan and Swarna was observed, where Moroberekan was at the positive side of the two axes and Swarna was at the negative side. In order to further understand the effect of the individual traits on yield stability across lowland drought stress and non-stress and direct-seeded non-stress conditions, we calculated the percentage difference for each trait for 25 lines with highest mean yield and 25 with lowest mean yield across the three experiments (Additional file 8). Differences ranged from positive to negative in the trait groups except for traits related to yield potential where high-yielding lines had higher means for all traits. The analysis also showed the magnitude and direction of effect of different traits on yield stability across ecosystems. While the two groups of lines were highly contrasting for some drought-related traits such as bleeding rate, reduction of NDVI, and leaf: stem ratio, they showed very little difference for the other traits such as stem strength and stem diameter. However, a large proportion of traits across these trait groups showed intermediate level differences, indicating their importance in determining yield stability along with the traits that showed larger differences.

### Genetic analysis

#### CIM analysis with individual traits

**Fig. 5:**
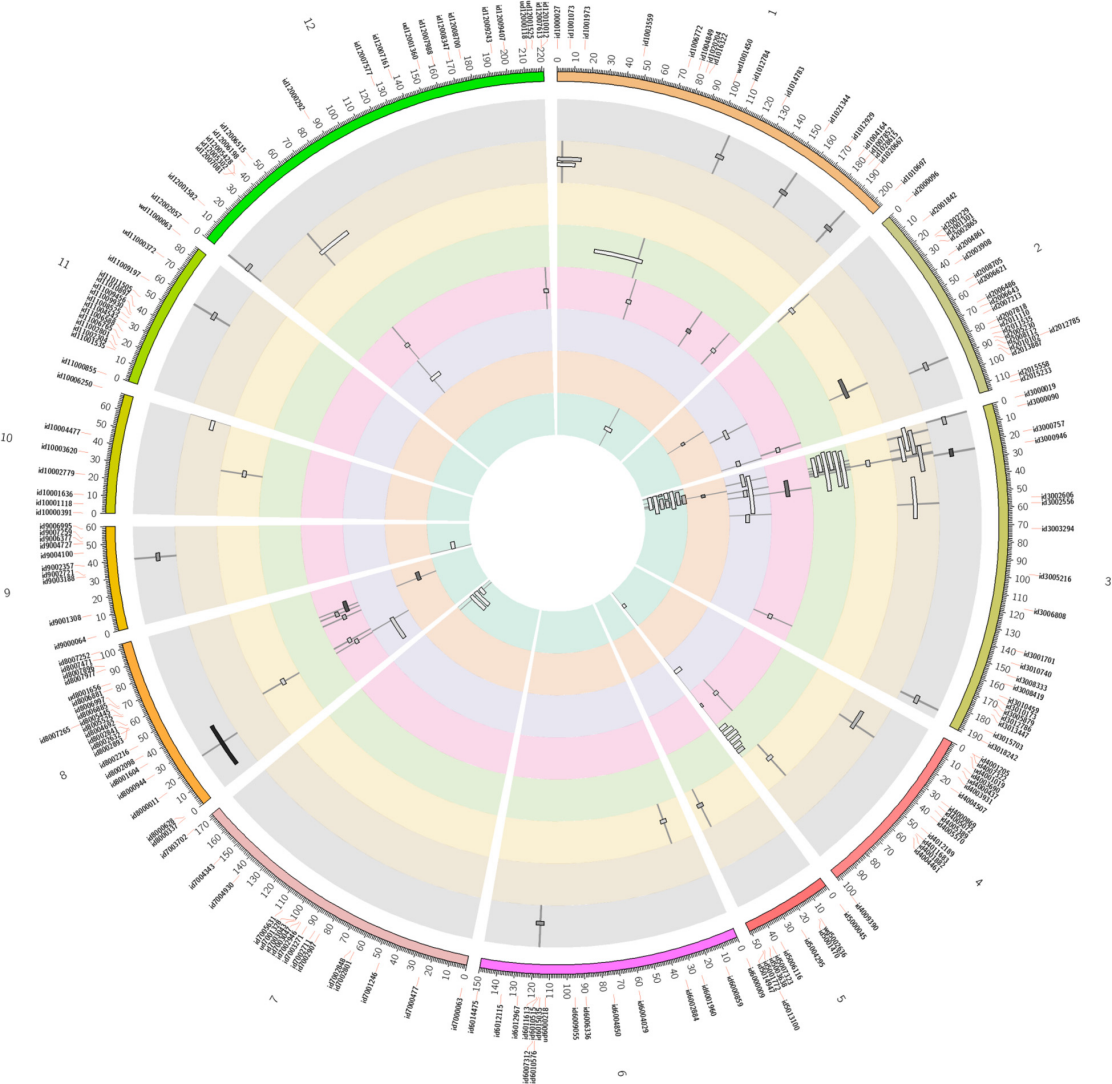
Circle plot showing the location of QTLs affecting single and composite traits identified through CIM and MLSIM analysis respectively. Colored bars showing the twelve rice chromosomes form the outermost circle, marker names (starting with the term ‘id/wd/ud’ followed by the number) and positions (cM) are presented along the chromosomes. Colored concentric circles sequentially from the center represent the QTLs for drought tolerance (CIM), QTLs for drought tolerance (MLSIM), QTLs for yield potential (CIM), QTLs for yield potential (MLSIM), QTLs for lodging resistance (CIM), QTLs for lodging resistance (MLSIM), QTLs for adaptation to direct seeding (CIM) and QTLs for adaptation to direct seeding (MLSIM). Horizontal bars within the rings represent the QTL span while vertical lines represent the peak position. The intensity of color of QTL bars shows the amount of variance explained by the QTL with color intensity increasing with QTL effect

A total of 49 QTLs were identified through CIM analysis for the four trait groups (Additional file 9). The QTLs were distributed across nearly all chromosomes with the highest densities observed on chromosomes 3, 4, and 7 (Fig. 5). In particular, QTLs for traits across all four trait groups were identified on chromosome 3 close to the *qDTY*_*3.2*_ region. For the drought tolerance trait group, QTLs were seen for traits related to drought and grain yield. However, higher numbers of QTLs were identified for drought-related traits as compared to yield-related traits. QTL clusters were observed at chromosome 3 at the *qDTY*_*3.2*_ region, including a QTL for grain yield under drought. However, the yield-enhancing allele in this case came from the susceptible parent. While this QTL is known to affect the flowering time along with its effect on grain yield under drought, the staggered seeding of the progeny from different maturity groups may explain Swarna’s contribution of the yield-enhancing allele at this locus. The advantage of having the Moroberekan allele at this locus can be seen through its effect on several other drought-related traits affecting plant function (Additional file 9). Apart from chromosome 3, another QTL cluster was observed at chromosome 7 where root mass, sap from the root system, and canopy temperature-related QTLs were identified (Additional file 9, Fig. 5). Other QTLs on chromosome 1, 4, and 9 were identified for root mass density, nodal root number, and panicle length at harvest. Similar to drought tolerance, QTLs for traits related to yield potential were contributed by both parents. However, QTLs for grain yield *per se* were not identified. The high-yielding parent Swarna contributed to QTLs for number of panicles and tillers at harvest on chromosomes 3 and 4, respectively. It also contributed to two QTLs on chromosomes 3 and 12 for plant height. The donor parent Moroberekan also contributed to several QTLs related to yield potential, including QTLs for shoot biomass, harvest index, and panicle length. Two major QTL clusters were identified on chromosomes 3 and 4 for traits related to lodging resistance. QTLs for the two major lodging-related traits – stem strength and diameter – were also located in these QTL clusters. The QTLs on chromosome 3 were contributed by Swarna while those on chromosome 4 were contributed by Moroberekan. Both QTL clusters showed consistent effects on lodging- related traits under upland direct-seeded and lowland transplanted conditions. QTLs were also identified for traits related to adaptation to direct seeding. In particular, QTLs for seedling emergence contributed by Moroberekan and Swarna were observed on chromosomes 1 and 3, respectively. Apart from this, some of the yield-related QTLs identified under transplanted lowland conditions also showed an effect under direct-seeded conditions. These included QTLs related to flowering time, plant height, and panicle length. A QTL for grain weight was also identified on chromosome 10.

### MLSIM analysis with composite traits

**Fig. 6:**
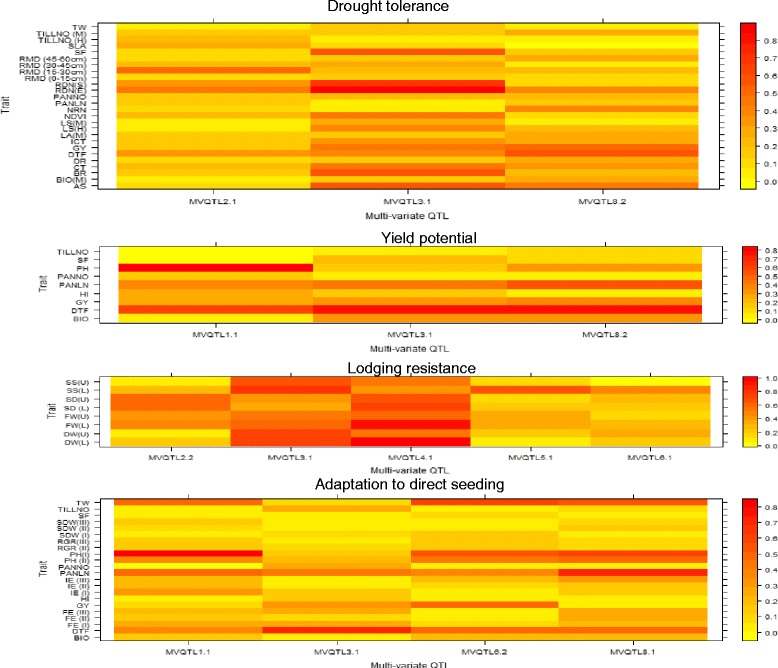
Heat maps showing the relative contribution of univariate traits to major MVQTLs identified for the four composite traits

Unlike CIM, MLSIM allowed the identification of QTLs for composite traits representing a group of individual traits such as drought tolerance, yield potential, lodging resistance, and adaptation to direct seeding. A total of 3, 15, 8, and 12 multivariate QTLs (MVQTLs) were identified for the four trait groups, respectively (Additional file 10). This method identified QTLs in some of the locations identified through CIM. For example, MVQTLs were identified on chromosome 3 for almost all trait groups, close to the positions where QTL clusters were identified through CIM at this locus. Similarly, chromosome 4 showed the presence of MVQTLs for lodging resistance close to the QTL cluster identified for the component traits at this locus. Furthermore, several other MVQTLs were observed for the four trait groups at the locations where the QTLs were not detected through CIM analysis (Fig. 5). In particular *MVQTL*_*8.1*_ and/or *MVQTL*_*8.4*_ were observed consistently across the four trait groups. However, the CIM analysis did not detect these two loci with such high consistency. The contribution of different traits to different MVQTLS was also assessed through this analysis which can help select these QTLs based on the traits affected for further utilization in breeding programs (Fig. 6, Additional file 11). This helped in the classification of QTLs into two specific classes: (1) those influencing the majority of the traits (such as *MVQTL*_*3.1*_ for drought tolerance and *MVQTL*_*3.1*_ and *MVQTL*_*4.1*_ for lodging resistance), and (2) those influencing few specific traits (such as *MVQTL*_*2.1*_ for drought and *MVQTL*_*2.2*_ for lodging resistance). While class 1 MVQTLs were observed for the trait groups on drought tolerance and lodging resistance, class 2 MVQTLs were observed for the trait groups on yield potential and adaptation to direct seeding.

### Epistatic interactions

In addition to beneficial alleles contributed by both parents, epistatic interactions among loci may also be the cause of transgressive segregation in the progeny. In this study, epistasis was observed for all four trait groups, particularly for traits related to lodging resistance and adaptation to direct seeding. Interactions were seen for stem diameter and shoot dry weight per plant for lodging resistance, and panicle length and first emergence for adaptation to direct seeding. Epistatic interactions were also observed for bleeding rate and biomass for drought and yield potential, respectively. *eQTL*_*1.1*_*, eQTL*_*3.1*,_ and *eQTL*_*3.2*_ were the three most consistent loci showing epistatic interactions with different loci across the genome (Additional file 12).

## Discussion

We studied traits related to drought tolerance, yield potential, lodging resistance and adaptation to direct seeding to understand trait interaction, and mapping and aligning QTLs. Traits were targeted individually and in groups for statistical and genetic analysis with the aim of understanding the basis of the rice crop’s adaptation to varying environmental conditions to which it can be exposed. The parents Moroberekan and Swarna proved to be specifically suitable for studying such a wide range of traits (Fig. 1). The high contrast among these two cultivars allowed us to achieve high variation in the mapping population for the majority of the traits (Fig. 2). Rice is cultivated in a much wider range of environments compared to any other cereal crop. This has allowed high genetic variation to develop in rice cultivars for a wide range of traits. Our study provides further evidence for this, where crossing two cultivars provided substantial genetic variation in the population for a wide range of related and unrelated traits.

The traits in this study grouped into three specific clusters in MDS analysis based on their correlations within and across trait groups (Fig. 3). PCA confirmed these results, with Moroberekan and Swarna showing high contrast and the four trait groups showing similar patterns of arrangements as seen in the MDS analysis along PC1 and PC2, indicating the correlation within and across trait groups (Fig. 4). For example, traits related to adaptation to direct seeding showed correlations with several yield potential-related traits, as well as some traits related to drought tolerance. Such correlations indicate the interactions of plant type, phenology, yield potential, and drought tolerance to be affecting adaptation to direct seeding. The most direct evidence to this was the high positive correlation of grain yield under transplanted and direct-seeded non-stress conditions, and the correlation of seedling emergence and relative growth rate under direct-seeded conditions with root and shoot mass under drought. This pattern signifies that the specificity of traits required for efficient adaptation to direct seeding varies at different growth stages of the crop. Further evidence of this comes from several recently-developed drought-tolerant varieties that show better adaptation to direct seeding compared to high-yielding varieties developed specifically for irrigated conditions [16]. While these varieties were developed through selection for yield under drought, combined with semi-dwarf plant type and high yield, the effect of selection for traits such as emergence and growth rate on adaptation to direct seeding is clear. Similarly, lodging-related traits showed correlations with plant type and other yield-related traits under direct-seeded and transplanted non-stress conditions. This indicates the role of a much wider range of traits in determining adaptation to lodging than traits that directly relate to it.

The trait interactions observed in the phenotypic analysis were also apparent in the QTL mapping. A QTL cluster was detected for drought-related traits on chromosome 3 which showed effects on a wide range of traits across the four trait groups (Fig. 5). Independent studies have shown the effect of this locus on traits such as grain yield under drought, lodging resistance, and yield- related traits [14, 17–19]. The locus also collocates with *HD9* which is a major gene for days to flowering. In this study, QTLs for lodging-related traits like stem diameter or stem strength and for drought tolerance such as NDVI, canopy temperature and bleeding rate were observed at this locus. Interestingly, grain yield correlated positively with NDVI but showed negative correlation with canopy temperature (Additional file 6). Better maintenance of canopy cover (high NDVI) and transpiration (related to low canopy temperature) are expected to be beneficial under drought and these traits collocated with *qDTY*_*3.2*_ may indicate better ability to access soil water. Although no QTLs for root traits were identified in this region, a positive correlation between grain yield and root mass density at the soil depth of 45 to 60 cm was observed. In addition, a negative correlation was observed between grain yield and bleeding rate which confirms earlier observations that drought-tolerant rice lines generally display low bleeding rate [20]. Apart from this, QTLs related to traits affecting adaptation to direct seeding were also observed, confirming the effect of this locus on a number of traits. Further, QTLs were contributed by both parents at this locus for different traits, indicating the linkage of drought-related genes at this locus. A high diversity of genes related to plant function under biotic and abiotic stresses has also been reported previously at this locus [17, 18, 21]. Another QTL cluster, including a QTL for root mass density at depth, canopy temperature, and absolute amount of sap, was detected on chromosome 7. QTLs for root-related traits such as root thickness and maximum root length have been reported previously close to this locus [22, 23]. Similarly, a QTL cluster close to a previously reported QTL for lodging resistance [14] was detected on chromosome 4. These QTL clusters could play an important role in improving rice for a wide range of traits through targeted alleleic introgression using marker-assisted selection. Our study also included some important and relatively newly researched traits related to drought such as canopy temperature and NDVI. While these traits are being increasingly used for high throughput phenotyping for drought tolerance [24], knowing the QTLs underlying them can be important in understanding their genetic control. Similarly, QTLs for new traits such as early and uniform emergence can be useful in improving crop establishment under direct-seeded conditions.

We employed the composite trait approach for identification of QTLs affecting trait groups through MLSIM analysis. This approach has been used successfully to identify multivariate QTLs controlling root architecture in rice [25]. Some of these QTLs co-localized with the QTLs identified for individual traits through the CIM analysis while some others were identified at distinct new positions (Fig. 5). For example, MVQTLs for all four composite traits were identified on chromosome 8 while few QTLs for individual traits were observed on this chromosome. Hence the analysis allowed us to better explain the genetic control of these traits through identification of regions that were undetected by single-trait-based approaches. In addition, the analysis also allowed us to understand the effect of different traits on different MVQTLs (Fig. 6). The distinct pattern of correlation of these QTLs with the underlying traits helps in understanding their possible utilization in breeding programs. Some of these QTLs that affect the majority of the underlying traits need to be carefully used based on the allelic influences on different underlying traits, while the other QTLs with effects on specific traits can be incorporated in breeding programs more easily for marker-assisted selection. While transgressive segregants were observed in the progeny, the presence of epistatic interaction is apparent. Epistatic interactions were observed for traits related to all four trait groups in this study. Epistasis has been reported previously for complex traits such as yield under drought stress [9, 17, 26] and non-stress conditions [12], however those for lodging-related traits (stem diameter in this case) have not been reported in rice to the best of our knowledge.

The phenotypic and genetic analysis of our study focused not only on individual traits but also on trait groups as a whole which enabled us to better understand the basis of yield stability of the rice crop across different ecosystems. Alignment of QTLs for a wide range of traits can also be achieved through meta-analysis; however most studies of this nature have been dealing with traits related to a particular target [21, 23, 27, 28]. Both the CIM and MLSIM QTL mapping methods have allowed us to identify and align QTLs with effects on a wide range of traits related to adaptation to multiple establishment and growth conditions, which can provide an advantage to the breeding programs targeting varying environments. This study also allowed us to understand the interactions between traits belonging to four very distinct and important trait groups that play crucial roles in the adaptation of rice plants to varying environments. Results from this study have allowed us to further understand the genetic and physiological basis of adaptation of rice to a wide range of environments.

## Conclusion

Our study targeted traits belonging to four diverse trait groups to understand correlations among these traits as well as to detect and align QTLs for them. Significant correlations between traits within and across trait groups were observed. The study identified component traits leading to better performance of genotypes under varying ecosystems and cultivation practices, and successfully identified and aligned QTLs on the rice genome belonging to four trait groups and their component traits. The highest numbers of QTLs were located on chromosome 3. The QTLs identified in this study can be used for targeted trait improvement following marker assisted breeding to develop rice lines with wider adaptation and yield stability across environments and cultivation practices.

## Methods

### Plant materials

A mapping population of 250 BC_2_F_3_-derived lines developed from the cross Moroberekan/3* Swarna was used in this study. Moroberekan, the tolerant donor, is an upland- adapted *tropical japonica* [29] landrace from New Guinea. It is a long-duration cultivar with sturdy plant type, deep roots, and is tolerant to drought and rice blast. However, this variety has poor yield potential because of its low tillering ability and lower number of grains per panicle. On the other hand, Swarna (MTU 7029), the drought-susceptible recipient parent, is a lowland-adapted high-yielding *indica* variety [30, 31] derived from the cross Vashishtha X Mahsuri. It is a long-duration semi-dwarf variety with high tillering ability and grain yield. This variety is grown on a large area in rainfed and irrigated ecologies across India, Nepal, and Bangladesh and is regarded as a mega-variety of rice.

### Experimental conditions and field management

Four field experiments were conducted in upland and lowland conditions at the experiment station of the International Rice Research Institute (IRRI), Los Baños, Laguna, Philippines (14°11′N, 121° 15′E) in the dry season (DS) and wet season (WS) of 2013 (Additional file 13). Throughout the study, the term ‘upland’ is used for field experiments conducted under direct-seeded, non-flooded, aerobic conditions while the term ‘lowland’ refers to field experiments conducted under flooded, puddled, transplanted and anaerobic conditions. Experiment 1 (1A, 1B, and 1C) was conducted under reproductive-stage drought stress conditions with early-, medium-, and late-maturing lines, respectively, due to heterogeneity in maturity duration in the population and with the aim of applying drought stress at the reproductive stage. Experiments 2 and 3 were conducted under non-stress conditions in lowland and upland, respectively, and Experiment 4 was conducted under upland seedling-stage drought stress. Experiments 2–4 were conducted with the full set of 250 lines and had no groupings based on maturity. All experiments were conducted in an α lattice design with three replicates each for Experiments 1 and 2 and two replicates each for Experiments 3 and 4 (Additional file 13).

In the lowland experiments, lines were grown in a wet bed nursery for 21 days before being transplanted in fields that were kept well-watered up to a month after transplanting. A spacing of 20 and 25 cm was maintained between plants and rows, respectively, with two to three seedlings transplanted per hill. In the upland experiments, lines were direct seeded in non-puddled soil at a density of 2.0–2.5 g m^−1^ and a depth of approximately 3 cm with a row spacing of 25 cm. Fields were sprinkler-irrigated to initiate seed germination and were surface-irrigated starting at one week after seedling emergence to maintain lowland-like conditions. All control treatments were irrigated 2–3 times per week throughout the crop duration. The stress experiments were also irrigated 2–3 times per week during crop establishment and early vegetative growth, and the drought stress treatment was initiated by withholding irrigation starting from 45 to 75 days after sowing (DAS), depending on the maturity group (Additional file 13). In Experiment 4, no irrigation was provided up to 21 DAS, after which full emergence was observed in all plots. The field was then re-irrigated and maintained well-watered until crop maturity. Complete fertilizer (14-14-14) was applied 13 days after transplanting in Experiments 1 and 2 (both the stress and control treatments) at a rate of 45 kg NPK ha^−1^, and a second application as topdressing was made before panicle initiation using ammonium sulfate at a rate of 45 kg N ha^−1^. Experiment 3 received 45 kg NPK ha^−1^ at 7 DAS, followed by topdressings of 45 kg N ha^−1^ on 34 and 38 DAS. No fertilizer was applied to Experiment 4. Manual weeding was done regularly in all experiments.

### Phenotypic data collection

Traits related to drought tolerance (27 traits), yield potential (9 traits), lodging resistance (8 traits), and adaptation to direct seeding (22 traits) were recorded (Additional file 14).

### Trait group 1: drought tolerance

Traits related to the ability to maintain shoot and root growth, water uptake, flowering, and grain yield under drought were classified in Trait group 1: Drought tolerance. These traits were recorded under lowland reproductive stage drought-stress conditions (Experiments 1A–C).

Shoot growth and groundcover dynamics were monitored according to NDVI measured around mid-day using a Greenseeker Hand-held Sensor (NTech Industries, CA, USA). Canopy temperature was measured at three locations per plot using a hand-held data-logging infrared (IR) sensor (Apogee Instruments, Logan UT, USA) after stress initiation. The increase in canopy temperature throughout the stress period was calculated as the slope (*X*) of the following equation:

$$ CT=\left(ICT\ X\ d\right)+b $$

where, *CT* is the canopy temperature, *ICT* is the increase in canopy temperature, d is the days after stress initiation, and b is the y intercept.

Root samples were taken 2–3 days after re-watering following severe stress symptoms at the grain-filling stage using a 4 cm-diameter core sampler (fabricated at IRRI, Los Baños, Philippines) to a depth of 60 cm. Soil cores were divided into 15-cm segments, and roots were washed by repeatedly mixing the soil with water in a container, and pouring the root-water suspension over a 1-mm plastic sieve. Only roots identified as living rice roots were retained for measurement. All samples were dried and weighed. Root mass density was calculated as the root mass per 15-cm soil core segment divided by the volume of the soil core segment. Percentage of deep roots was calculated as the mass of roots in the core below 30 cm divided by the total mass of roots inside the core. Bleeding rate measurements were carried out as described by Morita and Abe [32] and measured at mid-stress when soil water tension fell below −30 kPa. Sap exuded from the root zone was quantified in one hill per plot in both control and drought treatments. Starting at 7:00 am, shoots were cut at ~15 cm from the soil surface, and cut stems connected to the undisturbed root system were wrapped in a 625 cm^2^ cotton towel, then covered with a polyethylene bag, sealed at the base with a rubber band, and left for 4 h to absorb xylem sap that flowed from the cut stems. The towel, bag, and rubber band used for each hill were weighed before use. After 4 h, the bags and towels were removed from the stems, sealed, and immediately weighed to quantify the bleeding rate from the intact root system. Leaves from each hill were collected and kept inside a cold box for leaf area measurement. Tiller number was counted and leaves were separated from the stem and were dried and weighed to determine the biomass and leaf:stem ratio at mid-stress. In order to account for variation in plant size within and among genotypes, all sap exudation values were normalized by the dry shoot biomass of the hill from which sap was collected to calculate the bleeding rate. Specific leaf area was calculated as the leaf area, measured using a roller-belt-type leaf area meter (Li-Cor, Model LI-3100C, Li-Cor, Lincoln, NE, USA) divided by the leaf dry weight. Days to flowering (DTF) was recorded when about 50 % of the plants in the plot had flowered. Plant height (PH) of three plants from each plot was measured at maturity from ground level to the tip of the tallest tiller and averaged to get the mean PH for analysis. At physiological maturity, three hills were sampled in each plot for the measurements of the yield components including number of tillers and panicle, panicle length (cm), spikelet fertility (%), 1000-grain weight (gm) and rachis-stem-leaf dry weight (gm). Grain yield was measured from a sampled area of 1.5 m^2^ and dried to 14 % moisture content. The weight of grains was then used to calculate the kg ha^−1^ yield for each plot for further analysis.

### Trait group 2: yield potential

Grain yield (kg ha^−1^) and yield-related traits such as number of panicles and tillers at harvest, spikelet fertility percentage (by weight), panicle length (cm), biomass (kg ha^−1^), DTF and plant height (cm) were classified in Trait group 2: Yield potential. These traits were recorded under lowland well-watered conditions (Experiment 2). The harvested area for grain yield was 1.5 m^2^.

### Trait group 3: lodging resistance

Traits related to lodging resistance were measured under lowland and upland non-stress conditions (Experiments 2 and 3, respectively). These included stem strength, stem thickness, and fresh and dry weight per plant. Stem strength was measured using the prostrate tester (Daiki Rika Kogyou Co., Tokyo). All data were recorded from three plants from each plot. At physiological maturity, plants were cut off at 40 cm height, with the prostrate tester set perpendicularly at the middle (20 cm), and the pushing resistance of the lower part of the plant was measured by pushing the plants to the point at which the stem broke and the scale displacement (mm) due to pushing resistance was recorded. The stem diameter was measured from the same three plants at a height of 40 cm using a screw gauge. The plants were then harvested from the base to measure fresh weight per plant and then oven dried for three days at 70 °C and weighed to estimate the dry weight per plant. Average values for all parameters were calculated and used for further analysis.

### Trait group 4: adaptation to direct seeding

Visual observations of the time (DAS) to first emergence (when the first seedlings of a plot emerged) and full emergence (most of the seedlings in each plot had emerged) were recorded in the seed bed nursery of Experiment 1 and of the direct-seeded experiments (3 and 4). Shoots from five seedlings per plot were sampled at a two-week interval (18 DAS and 32 DAS) to determine the relative growth rate (RGR) in Experiment 2. Shoots were dried and weighed to determine the biomass for each sampling date and the RGR was calculated as:

$$ RGR=\frac{\left[ \ln (B2)- \ln (B1)\right]}{\left(D2-D1\right)} $$

Where, *B2* is the shoot biomass on date 2, *B1* is the shoot biomass on date 1, *D2* is date 2 and *D1* is date 1.

In addition, grain yield and traits related to yield potential and phenology under direct-seeded conditions such as DTF, panicle number at harvest, tiller number at harvest, spikelet fertility, 1000-grain weight, and panicle length were recorded in Experiment 3. The harvested area for grain yield in Experiment 3 and 4 was 1 and 0.25 m^2^, respectively.

### Statistical analysis

Statistical analysis for the computation of means and standard error of difference (SED) were conducted using CROPSTAT version 7.2.3. A mixed model analysis of data from individual years was carried out using the model:

$$ {y}_{ijk}=\mu +{g}_i+{r}_j+{b}_k\left({r}_j\right)+{e}_{ijk} $$

where y_ijk_ is the measurement recorded in a plot, *μ* is the overall mean, *g*_*i*_ is the effect of the i^th^ genotype, *r*_*j*_ is the effect of the j^th^ replicate, *b*_*k*_*(r*_*j*_*)* is the effect of the k^th^ block within the j^th^ replicate, and *e*_*ijk*_ is the error. Genotypic effects were considered fixed and the replicates and block effects were random.

Correlations between the traits were estimated using the ‘cor’ function in R 3.1.0 [33]. For better visualization, the distance matrix was calculated using the correlation values between the traits and were used to conduct a MDS analysis using STAR (version 2.0.1). To perform PCA, all traits were first standardized to a mean of zero and standard deviation of one, and missing values were filled with zero (population mean). PCA was calculated with the prcomp function in R [33].

### Genotypic data

Fresh leaves for all lines were collected and freeze-dried. DNA was extracted from the freeze-dried leaf samples by a modified CTAB method in deep-well plates. The DNA was then quantified and purified and lines were genotyped using KASPar SNP assays. These SNPs were selected as subsets from the set of 1536 and 44 K SNP chips [34, 35], converted to SNP assays and made available through the integrated breeding platform (https://www.integratedbreeding.net/482/communities/genomics-crop-info/crop-information/gcp-kaspar-snpmarkers). A total of 2015 SNP markers were screened for polymorphism between the two parents. Out of these 2015 SNPs, 591 polymorphic SNP loci were identified. The genotypic data from a set of 193 polymorphic SNP markers was used to generate the genotypic profile of the population.

### Genetic analysis

Composite interval mapping (CIM) was conducted using QTL Network 2.1 [36] based on a mapping methodology outlined by Yang et al. [37]. Putative regions within the QTLs were identified with this software based on a one-dimensional genome scan taking selected candidate intervals as cofactors. A mixed linear model framework was used to perform the mapping procedure with an F-statistic based on Henderson method III for hypothesis testing. A total of 1,000 permutation tests were used to minimize the genome-wise type I error and to calculate the critical F-value. Apart from the CIM analysis, MLSIM analysis using the procedure detailed in Anderson et al. [38] was also conducted to identify putative QTLs controlling multiple traits simultaneously. Briefly, the QTL allele frequency (Pqm) conditional on the flanking marker genotypes for each point in the genome of each line was calculated. These Pqm values were then used as predictors [39] for multivariate analysis of variance (MANOVA) across the genome. Statistical significance was determined with randomization tests using 1,000 permutations. The identification of multiple QTLs was conducted sequentially, each conditional on all previously identified QTLs until no further significant QTL was found. To further understand QTL effects, a one-dimensional ‘composite trait’ was also calculated for each QTL by identifying the linear trait combination best explained by QTL genotypes with discriminant function analysis. Circle plot showing the chromosome map and QTLs was developed using Circos [40].

### Availability of supporting data

The data sets supporting the results of this article are included within the article and its additional files.

## Additional files

Additional file 1:
**Diversity of Moroberekan and Swarna for major traits related to drought tolerance, yield potential, lodging resistance, and adaptation to direct seeding.**
Additional file 2:
**Analysis of variance table for lowland drought stress experiments conducted with early (E), medium (M), and late (L) duration lines including means of parents and progenies and P values.** NS: Non-significant, NA: Data not available, a: probability of difference between genotypes *, **, ***, **** significant at 5, 1, 0.1, 0.01 % P levels, respectively, NDVI: normalized difference vegetation index. Additional file 3:
**Analysis of variance table for lowland well-watered experiment including means of parents and progenies and P values.** NS: Non-significant, a: probability of difference between genotypes *, **, ***, **** significant at 5, 1, 0.1, 0.01 % P levels, respectively. Additional file 4:
**Analysis of variance table for upland well-watered experiments including means of parents and progenies and P values.** NS: Non-significant, a: probability of difference between genotypes *, **, ***, **** significant at 5, 1, 0.1, 0.01 % P levels, respectively. Additional file 5:
**Analysis of variance table for upland seedling stage drought experiments including means of parents and progenies and P values.** NS: Non-significant, a: probability of difference between genotypes *, **, ***, **** significant at 5, 1, 0.1, 0.01 % P levels, respectively. Additional file 6:
**Phenotypic correlation between 66 traits studied across the four trait groups: drought tolerance, yield potential, lodging resistance and adaptation to direct seeding.** Red and green colored cells represent values significant at 5 % and 1 % P levels respectively. Additional file 7:
**Percentage variation explained by principal components (PC) 1–65 in the population.**
Additional file 8:
**Percentage difference between means of trait values of 25 high and low yielding lines from the population.** Traits with red bars show higher values for low yielding lines while those with blue bars show higher values for high yielding lines. Additional file 9:
**QTLs for traits related to drought tolerance, yield potential, lodging resistance and adaptation to direct seeding identified through CIM in DS and WS 2013.** A = additive effect, R^2^ = percentage of phenotypic variance explained by the QTL. Additional file 10:
**QTLs for composite traits drought tolerance, yield potential, lodging resistance and adaptation to direct seeding identified through MLSIM analysis.** R^2^ = percentage of phenotypic variance explained by the QTL. Additional file 11:
**Contribution of univariate traits to MVQTLs detected for the four composite traits.** Names for each trait (numbered) in this table can be found in Additional file 14. Additional file 12:
**Epistatic interactions identified for traits related to the four trait groups.** AA: additive effect, R^2^: Phenotypic variance explained. Additional file 13:
**Details of experiments conducted in the 2013 DS and WS under lowland transplanted drought stress and non-stress and upland direct-seeded drought stress and non-stress conditions.**
Additional file 14:
**Traits studied under the four trait groups (drought tolerance, yield potential, lodging resistance, and adaptation to direct seeding) with trait codes and ecosystems.**

## Abbreviations

CIM: Composite Interval Mapping
MLSIM: Multivariate Least Square Interval Mapping
NDVI: Normalized Difference Vegetation Index
MDS: The multidimensional scaling
PCA: Principal component analysis
MVQTLs: Multivariate QTLs
SNP: Single nucleotide polymorphism
MANOVA: Multivariate analysis of variance
RGR: Relative growth rate
DS: Dry season
WS: Wet season
