# Supplementary material for: Understanding rice adaptation to varying agro-ecosystems: trait interactions and quantitative trait loci
Source: BMC Genet. 2015 Aug 5;16:86. doi: 10.1186/s12863-015-0249-1 (PMC4526302; doi:10.1186/s12863-015-0249-1)
Supplement: Additional file 1: — Diversity of Moroberekan and Swarna for major traits related to drought tolerance, yield potential, lodging resistance, and adaptation to direct seeding. [file 12863_2015_249_MOESM1_ESM.docx]

**Additional file 1:** Diversity of Moroberekan and Swarna for major traits related to drought tolerance, yield potential, lodging resistance, and adaptation to direct seeding.

| Trait | Well watered | |  | Drought | |  | Direct seeded | |
| --- | --- | --- | --- | --- | --- | --- | --- | --- |
|  | Moroberekan | Swarna |  | Moroberekan | Swarna |  | Moroberekan | Swarna |
| Plant height (cm) | 112.0 | 82.4 |  | 101.6 | 53.8 |  | 105.8 | 77.1 |
| Days to flowering | 92.9 | 93.6 |  | 95.3 | 99.0 |  | 94.9 | 98.5 |
| Number of tiller m^-2^ at harvest | 184.1 | 415.9 |  | 105.9 | 303.3 |  | 125.6 | 302.2 |
| Number of panicle m^-2^ at harvest | 149.2 | 380.2 |  | 57.6 | 231.2 |  | 99.2 | 274.6 |
| Spikelet fertility (Percentage by weight) | 96.8 | 96.5 |  | 71.5 | 41.1 |  | 94.9 | 94.7 |
| Panicle length at harvest (cm) | 23.8 | 20.8 |  | 18.2 | 18.0 |  |  |  |
| Grain yield (kg ha^-1^) | 993.2 | 3322.7 |  | 179.7 | 310.6 |  | 953.2 | 4591.2 |
| Root mass density (0-15 cm) |  |  |  | 0.1659 | 0.6285 |  |  |  |
| Root mass density (15-30 cm) |  |  |  | 0.1006 | 0.1017 |  |  |  |
| Root mass density (30-45 cm) |  |  |  | 0.0818 | 0.0162 |  |  |  |
| Root mass density (45-60 cm) |  |  |  | 0.0007 | 0.0004 |  |  |  |
| Percentage deep roots |  |  |  | 11.5 | 3.0 |  |  |  |
| Nodal root number | 20.6 | 27.5 |  |  |  |  |  |  |
| Stem diameter (mm) | 5.9 | 3.5 |  |  |  |  | 6.2 | 3.1 |
| Stem strength | 31.1 | 16.6 |  |  |  |  | 27.4 | 14.2 |
| Full emergence |  |  |  |  |  |  | 11.1 | 7.0 |
