# Supplementary material for: Understanding rice adaptation to varying agro-ecosystems: trait interactions and quantitative trait loci
Source: BMC Genet. 2015 Aug 5;16:86. doi: 10.1186/s12863-015-0249-1 (PMC4526302; doi:10.1186/s12863-015-0249-1)
Supplement: Additional file 2: — Analysis of variance table for lowland drought stress experiments conducted with early (E), medium (M), and late (L) duration lines including means of parents and progenies and P values. NS: Non-significant, NA: Data not available, a: probability of difference between genotypes *, **, ***, **** significant at 5, 1, 0.1, 0.01 % P levels, respectively, NDVI: normalized difference vegetation index. [file 12863_2015_249_MOESM2_ESM.docx]

**Additional file 2:** Analysis of variance table for lowland drought stress experiments conducted with early (E), medium (M), and late (L) duration lines including means of parents and progenies and P values.

| **Trait name** | **Experiment 1(A)** | | | | **Experiment 1(B)** | | | | **Experiment 1(C)** | | | |
| --- | --- | --- | --- | --- | --- | --- | --- | --- | --- | --- | --- | --- |
|  | **Mean** | | | ***P^a^*** | **Mean** | | | ***P*** | **Mean** | | | ***P*** |
|  | **Progeny** | **Moroberekan** | **Swarna** |  | **Progeny** | **Moroberekan** | **Swarna** |  | **Progeny** | **Moroberekan** | **Swarna** |  |
| Plant height (cm) | 73 | 102 | 54 | **** | 73 | 91 | 54 | **** | NA | NA | NA | NA |
| Increase of canopy temperature | 0.33 | 0.34 | 0.37 | **** | 0.69 | 0.65 | 0.72 | **** | 0.33 | 0.30 | 0.34 | ** |
| Canopy temperature | 36 | 37 | 37 | **** | 34 | 35 | 34 | * | 34 | 33 | 35 | *** |
| Reduction of NDVI (early stress) | -0.004 | -0.01 | -0.004 | NS | -0.003 | -0.004 | 0.00003 | NS | 0.0 | 0.01 | 0.01 | NS |
| Reduction of NDVI (severe stress) | -0.02 | -0.02 | -0.03 | NS | -0.001 | 0.003 | -0.004 | NS | 0.0 | -0.05 | -0.03 | NS |
| NDVI | 0.40 | 0.40 | 0.37 | NS | 0.58 | 0.59 | 0.56 | NS | 0.37 | 0.33 | 0.42 | NS |
| Absolute amount of sap | 2.4 | 2.3 | 2.3 | NS | 1.5 | 1.6 | 1.4 | **** | 1.2 | 1.1 | 1.3 | *** |
| Bleeding rate (g sap g^-1^ shoot) | 0.26 | 0.34 | 0.34 | **** | 0.14 | 0.19 | 0.15 | **** | 0.10 | 0.08 | 0.10 | **** |
| Number of tillers m^-2^ (mid-stress) | 14 | 5 | 14 | **** | 12 | 5 | 15 | **** | 12 | 6 | 15 | *** |
| Shoot biomass at mid-stress | 11 | 7 | 8 | **** | 12 | 10 | 9 | ** | 14 | 14 | 14 | **** |
| Leaf area at mid-stress | 1098 | 540 | 1103 | **** | 917 | 652 | 1027 | **** | 900 | 680 | 1095 | **** |
| Leaf:Stem ratio at mid-stress | 3 | 2 | 5 | **** | 1 | 2 | 2 | **** | 1 | 1 | 1 | **** |
| Specific leaf area at mid-stress | 169 | 110 | 188 | **** | 152 | 109 | 167 | **** | 151 | 94 | 158 | *** |
| Days to flowering | 81 | 95 | 99 | **** | 86 | 95 | 93 | **** | 88 | 93 | 92 | **** |
| Root mass density (0-15 cm) | 0.4585 | 0.1659 | 0.6285 | NS | 0.3957 | 0.3141 | 0.3024 | NS | 0.4105 | 0.1629 | 0.2332 | *** |
| Root mass density (15-30 cm) | 0.1133 | 0.1006 | 0.1017 | NS | 0.0902 | 0.1614 | 0.0712 | * | 0.0827 | 0.0629 | 0.1329 | NS |
| Root mass density (30-45 cm) | 0.0257 | 0.0818 | 0.0162 | NS | 0.0266 | 0.0408 | 0.0242 | NS | 0.0255 | 0.0251 | 0.0286 | NS |
| Root mass density (45-60 cm) | 0.0032 | 0.0007 | 0.0004 | NS | 0.0047 | 0.0112 | 0.0003 | ** | 0.0056 | 0.0027 | 0.0037 | NS |
| Percentage deep roots | 5 | 11 | 3 | * | 6 | 6 | 7 | NS | 6 | 8 | 5 | NS |
| Grain yield (kg ha^-1^) | 619 | 180 | 311 | **** | 1680 | 950 | 2136 | **** | 1910 | 1215 | 2363 | **** |
| Number of tiller m^-2^ at harvest | 300 | 106 | 303 | **** | 257 | 119 | 297 | **** | 280 | 114 | 356 | **** |
| Number of panicle m^-2^ at harvest | 204 | 58 | 231 | **** | 218 | 92 | 259 | **** | 241 | 95 | 286 | **** |
| Spikelet fertility (Percentage by weight) | 59 | 72 | 41 | **** | 62 | 67 | 63 | *** | 84 | 90 | 86 | **** |
| Weight of 1000 grains (g) | 16 | 25 | 13 | **** | 19 | 28 | 17 | **** | 19 | 30 | 17 | **** |
| Panicle length at harvest (cm) | 18 | 18 | 18 | **** | 19 | 21 | 18 | **** | 19 | 20 | 18 | **** |
| Leaf:stem ratio at harvest | 0.8 | 0.6 | 0.8 | ** | 0.7 | 0.6 | 0.9 | **** | 0.6 | 0.6 | 0.7 | **** |

NS: Non-significant, NA: Data not available, a: probability of difference between genotypes *, **, ***, **** significant at 5, 1, 0.1, 0.01% P levels, respectively, NDVI: normalized difference vegetation index
