# Supplementary material for: Understanding rice adaptation to varying agro-ecosystems: trait interactions and quantitative trait loci
Source: BMC Genet. 2015 Aug 5;16:86. doi: 10.1186/s12863-015-0249-1 (PMC4526302; doi:10.1186/s12863-015-0249-1)
Supplement: Additional file 3: — Analysis of variance table for lowland well-watered experiment including means of parents and progenies and P values. NS: Non-significant, a: probability of difference between genotypes *, **, ***, **** significant at 5, 1, 0.1, 0.01 % P levels, respectively. [file 12863_2015_249_MOESM3_ESM.docx]

**Additional file 3:** Analysis of variance table for lowland well-watered experiment including means of parents and progenies and P values.

| **Trait name** | **Mean** | | | ***P^a^*** |
| --- | --- | --- | --- | --- |
|  | **Progeny** | **Moroberekan** | **Swarna** |  |
| Nodal root number | 26 | 21 | 28 | ** |
| Days to flowering | 86 | 93 | 94 | **** |
| Grian yield (kg ha^-1^) | 2704 | 993 | 3323 | **** |
| Plant height (cm) | 93 | 112 | 82 | **** |
| Shoot biomass (kg ha^-1^) | 12920 | 10951 | 14406 | ** |
| Number of tiller m^-2^ at harvest | 360 | 184 | 416 | **** |
| Number of panicle m^-2^ at harvest | 319 | 149 | 380 | **** |
| Harvest index | 0.36 | 0.25 | 0.42 | **** |
| Panicle length at harvest (cm) | 21 | 24 | 21 | **** |
| Spikelet fertility (Percentage by weight) | 95 | 97 | 97 | NS |
| Stem diameter (mm) | 4 | 6 | 3 | **** |
| Stem strength | 19 | 31 | 17 | **** |
| Dry weight per plant (g) | 220 | 376 | 185 | **** |
| Fresh weight per plant (g) | 380 | 707 | 313 | **** |
| Relative growth rate | 0.09 | 0.08 | 0.09 | ** |
| First emergence | 5 | 5 | 5 | **** |
| Full emergence | 8 | 11 | 8 | **** |

NS: Non-significant, a: probability of difference between genotypes *, **, ***, **** significant at 5, 1, 0.1, 0.01% P levels, respectively
