# Supplementary material for: Understanding rice adaptation to varying agro-ecosystems: trait interactions and quantitative trait loci
Source: BMC Genet. 2015 Aug 5;16:86. doi: 10.1186/s12863-015-0249-1 (PMC4526302; doi:10.1186/s12863-015-0249-1)
Supplement: Additional file 4: — Analysis of variance table for upland well-watered experiments including means of parents and progenies and P values. NS: Non-significant, a: probability of difference between genotypes *, **, ***, **** significant at 5, 1, 0.1, 0.01 % P levels, respectively. [file 12863_2015_249_MOESM4_ESM.docx]

**Additional file 4:** Analysis of variance table for upland well-watered experiments including means of parents and progenies and P values.

| **Trait name** | **Mean** | | | ***P^a^*** |
| --- | --- | --- | --- | --- |
|  | **Progeny** | **Moroberekan** | **Swarna** |  |
| Days to 50% flowering | 92 | 95 | 98 | **** |
| First emergence (DS2013) | 5 | 7 | 5 | **** |
| Full emergence (DS2013) | 8 | 11 | 7 | **** |
| Grain yield (kg ha^-1^) | 3223 | 953 | 4591 | **** |
| Plant height (cm) | 89 | 106 | 77 | **** |
| Shoot biomass (kg ha^-1^) | 8165 | 4687 | 7673 | ** |
| Number of tiller m^-2^ at harvest | 262 | 126 | 302 | *** |
| Number of panicle m^-2^ at harvest | 227 | 99 | 275 | *** |
| Harvest index | 0.35 | 0.30 | 0.39 | ** |
| Panicle length at harvest (cm) | 21 | 23 | 20 | **** |
| Spikelet fertility (Percentage by weight) | 95 | 95 | 95 | NS |
| Weight of 1000 grains (g) | 21 | 32 | 20 | **** |
| Stem thickness | 4 | 6 | 3 | **** |
| Stem strength | 19 | 27 | 14 | **** |
| Dry weight per plant | 207 | 174 | 170 | **** |
| Fresh weight per plant | 354 | 277 | 266 | **** |

NS: Non-significant, a: probability of difference between genotypes *, **, ***, **** significant at 5, 1, 0.1, 0.01% P levels, respectively
