# Supplementary material for: Understanding rice adaptation to varying agro-ecosystems: trait interactions and quantitative trait loci
Source: BMC Genet. 2015 Aug 5;16:86. doi: 10.1186/s12863-015-0249-1 (PMC4526302; doi:10.1186/s12863-015-0249-1)
Supplement: Additional file 5: — Analysis of variance table for upland seedling stage drought experiments including means of parents and progenies and P values. NS: Non-significant, a: probability of difference between genotypes *, **, ***, **** significant at 5, 1, 0.1, 0.01 % P levels, respectively. [file 12863_2015_249_MOESM5_ESM.docx]

**Additional file 5:** Analysis of variance table for upland seedling stage drought experiments including means of parents and progenies and P values.

| **Trait name** |  | **Mean** |  | ***P^a^*** |
| --- | --- | --- | --- | --- |
|  | **Progeny** | **Moroberekan** | **Swarna** |  |
| First emergence | 4.5 | 4.4 | 3.9 | NS |
| Full emergence | 8.6 | 8.5 | 8.4 | NS |
| Relative growth rate | 0.11 | 0.13 | 0.11 | NS |
| Plant height (cm) | 83.0 | 88.0 | 80.0 | NS |
| Shoot dry weight I | 0.11 | 0.11 | 0.11 | NS |
| Shoot dry weight II | 0.22 | 0.24 | 0.16 | NS |
| Shoot dry weight III | 0.71 | 1.05 | 0.71 | NS |

NS: Non-significant, a: probability of difference between genotypes *, **, ***, **** significant at 5, 1, 0.1, 0.01% P levels, respectively
