# Supplementary material for: Understanding rice adaptation to varying agro-ecosystems: trait interactions and quantitative trait loci
Source: BMC Genet. 2015 Aug 5;16:86. doi: 10.1186/s12863-015-0249-1 (PMC4526302; doi:10.1186/s12863-015-0249-1)
Supplement: Additional file 13: — Details of experiments conducted in the 2013 DS and WS under lowland transplanted drought stress and non-stress and upland direct-seeded drought stress and non-stress conditions. [file 12863_2015_249_MOESM13_ESM.docx]

**Additional file 13:** Details of experiments conducted in the 2013 DS and WS under lowland transplanted drought stress and non-stress and upland direct-seeded drought stress and non-stress conditions.

| **Experiment** | **Season** | **Stress condition** | **Population size** | **Experimental design** | **Replications** |
| --- | --- | --- | --- | --- | --- |
| 1A | DS2013 | Lowland, stress (early) | 72 | 6X12 AL | 3 |
| 1B | DS2013 | Lowland, stress (medium) | 72 | 6X12 AL | 3 |
| 1C | DS2013 | Lowland, stress (late) | 120 | 6X20 AL | 3 |
| 2 | DS2013 | Lowland, non-stress | 250 | 20X13AL | 3 |
| 3 | DS2013 | Upland, non-stress | 250 | 26X10AL | 2 |
| 4 | WS2013 | Upland, seedling stress | 250 | 26X10AL | 2 |
