# Supplementary material for: Understanding rice adaptation to varying agro-ecosystems: trait interactions and quantitative trait loci
Source: BMC Genet. 2015 Aug 5;16:86. doi: 10.1186/s12863-015-0249-1 (PMC4526302; doi:10.1186/s12863-015-0249-1)
Supplement: Additional file 14: — Traits studied under the four trait groups (drought tolerance, yield potential, lodging resistance, and adaptation to direct seeding) with trait codes and ecosystems. [file 12863_2015_249_MOESM14_ESM.docx]

**Additional file 14:** Traits studied under the four trait groups (drought tolerance, yield potential, lodging resistance, and adaptation to direct seeding) with trait codes and ecosystems.

| **Group** | **Trait** | **Trait code** | **Ecosystems** |
| --- | --- | --- | --- |
| **Group 1:** | Plant height (cm) | T1 | Lowland stress |
| **Drought tolerance** | Increase of canopy temperature (ºC d^-1^) | T2 | Lowland stress |
|  | Canopy temperature (ºC) | T3 | Lowland stress |
|  | Reduction of NDVI (early stress) | T4 | Lowland stress |
|  | Reduction of NDVI (severe stress) | T5 | Lowland stress |
|  | NDVI | T6 | Lowland stress |
|  | Absolute amount of sap (g) | T7 | Lowland stress |
|  | Bleeding rate (g sap g^-1^ shoot) | T8 | Lowland stress |
|  | Number of tillers m^-2^ (mid-stress) | T9 | Lowland stress |
|  | Shoot biomass at mid-stress (g) | T10 | Lowland stress |
|  | Leaf area at mid-stress (cm^2^) | T11 | Lowland stress |
|  | Leaf:Stem ratio at mid-stress | T12 | Lowland stress |
|  | Specific leaf area at mid-stress (cm^2^ g^-1^) | T13 | Lowland stress |
|  | Days to flowering | T14 | Lowland stress |
|  | Root mass density (0-15 cm; cm_root_ cm^-3^ _soil_) | T15 | Lowland stress |
|  | Root mass density (15-30 cm; cm_root_ cm^-3^ _soil_) | T16 | Lowland stress |
|  | Root mass density (30-45 cm; cm_root_ cm^-3^ _soil_) | T17 | Lowland stress |
|  | Root mass density (45-60 cm; cm_root_ cm^-3^ _soil_) | T18 | Lowland stress |
|  | Percentage deep roots | T19 | Lowland stress |
|  | Grain yield (kg ha^-1^) | T20 | Lowland stress |
|  | Number of tiller m^-2^ at harvest | T21 | Lowland stress |
|  | Number of panicle m^-2^ at harvest | T22 | Lowland stress |
|  | Percentage grain fertility (by weight) | T23 | Lowland stress |
|  | Weight of 1000 grains (g) | T24 | Lowland stress |
|  | Panicle length at harvest (cm) | T25 | Lowland stress |
|  | Leaf:Stem ratio at harvest | T26 | Lowland stress |
|  | Nodal root number | T27 | Lowland non-stress |
| **Group 2:** | Days to flowering | T28 | Lowland non-stress |
| **Yield potential** | Grain yield (kg ha^-1^) | T29 | Lowland non-stress |
|  | Plant height (cm) | T30 | Lowland non-stress |
|  | Shoot biomass (kg ha^-1^) | T31 | Lowland non-stress |
|  | Number of tiller m^-2^ at harvest | T32 | Lowland non-stress |
|  | Number of panicle m^-2^ at harvest | T33 | Lowland non-stress |
|  | Harvest index | T34 | Lowland non-stress |
|  | Panicle length at harvest (cm) | T35 | Lowland non-stress |
|  | Percentage grain fertility (by weight) | T36 | Lowland non-stress |
| **Group 3:** | Stem diameter (mm) | T37 | Upland |
| **Lodging resistance** | Stem strength (mm) | T38 | Upland |
|  | Dry weight per plant (g) | T39 | Upland |
|  | Fresh weight per plant (g) | T40 | Upland |
|  | Stem diameter (mm) | T41 | Lowland |
|  | Stem strength (mm) | T42 | Lowland |
|  | Dry weight per plant (g) | T43 | Lowland |
|  | Fresh weight per plant (g) | T44 | Lowland |
| **Group 4:** | Days to flowering | T45 | Upland |
| **DSR adaptation** | First emergence (DS2013) | T46 | Upland |
|  | Full emergence (DS2013) | T47 | Upland |
|  | Grain yield (kg ha^-1^) | T48 | Upland |
|  | Plant height (cm) | T49 | Upland |
|  | Shoot biomass (kg ha^-1^) | T50 | Upland |
|  | Number of tiller m^-2^ at harvest | T51 | Upland |
|  | Number of panicle m^-2^ at harvest | T52 | Upland |
|  | Harvest index | T53 | Upland |
|  | Panicle length at harvest (cm) | T54 | Upland |
|  | Percentage grain fertility (by weight) | T55 | Upland |
|  | Weight of 1000 grains (g) | T56 | Upland |
|  | First emergence (WS2013) | T57 | Upland |
|  | Full emergence (WS2013) | T58 | Upland |
|  | Relative growth rate (WS2013) | T59 | Upland |
|  | Plant height (cm, WS2013) | T60 | Upland |
|  | Relative growth rate (Lowland, DS2013) | T61 | Upland |
|  | First emergence (Lowland, DS2013) | T62 | Upland |
|  | Full emergence (Lowland, DS2013) | T63 | Upland |
|  | Shoot dry weight I (WS 2013) | T64 | Upland |
|  | Shoot dry weight II (WS 2013) | T65 | Upland |
|  | Shoot dry weight III (WS 2013) | T66 | Upland |
